# Enhancement of Energy Absorption Capability of 3D Printed Ti-6Al-4V BCC Lattice Structures by Adding Auxiliary Struts

**DOI:** 10.3390/ma18040732

**Published:** 2025-02-07

**Authors:** Jaryong Cho, Eunwoo Kim, Jeong Ho Kim, Chang-Yull Lee, Jin Yeon Cho

**Affiliations:** Department of Aerospace Engineering, Inha University, Incheon 22212, Republic of Korea; wkfyd94@inha.edu (J.C.); kimsilver1226@inha.edu (E.K.); jeonghokim@inha.ac.kr (J.H.K.); cylee@inha.ac.kr (C.-Y.L.)

**Keywords:** lattice structure, finite element method, energy absorption, crashworthiness, EBM metal 3D printing, quasi-static compression test

## Abstract

Lattice structures, composed of interconnected struts, offer an efficient way to reduce structural weight while maintaining structural integrity. Because of this potential, this work aims to investigate and develop an efficient variant form of a BCC (Body-Centered Cubic) lattice structure to enhance the structural robustness and energy absorption capability, based on the Maxwell stability criterion. And we specifically changed the bending-dominated to stretching-dominated behavior by adding auxiliary struts, according to the theory, and confirmed how this affects the compression behavior of the structure. For this purpose, horizontal auxiliary struts are added for the first time to the BCC structure along with vertical struts. As a macroscale cellular lattice structure, a unit cell size of 12 mm is considered. For the considered macroscale cellular lattice structures, FEA (finite element analysis) is employed to numerically investigate the stress distribution and compressive deformation mechanisms. Then, quasi-static compression tests are carried out to measure the energy absorption performance of the lattice structures manufactured by the EBM (Electron Beam Melting) metal additive manufacturing technique, which has advantages in building lattice structures without supporters. A comprehensive investigation reveals that a newly designed lattice structure offers significant advantages in structural robustness, with energy absorption capability increased by 365% compared to existing structures, achieved by incorporating vertical and cross-shaped horizontal auxiliary struts into the original BCC lattice configuration. The enhanced lattice structures can be utilized in industries where low-weight and high-strength are needed, such as aerospace, marine, and other industries.

## 1. Introduction

A lattice structure refers to a three-dimensional structure formed by connecting regularly arranged points in a crystal [1]. This lattice structure is a strut-based porous structure and has the advantage of being lighter weight than a general shell-designed or solid-designed structure. And, depending upon its internal connective structure, it can withstand a higher load than shell or solid structures under the same mass condition. Consequently, lattice structures have been investigated to be used not only in the construction field but also in the aerospace and marine industries, which require weight-lightened energy absorbing structures [2,3,4].

Among the many types of lattice structure, a BCC structure refers to a structure in which one point is arranged at each corner and the center of a cube. Then, connecting the center point and corner points brings out a BCC lattice structure [5,6,7,8]. Its shape viewed from three axes (*X*-axis, *Y*-axis, *Z*-axis) is the same as an X shape, and its arrangement is formed by repeating its structure throughout the crystal spatial pattern. Also, a BCC lattice structure is a kind of bending-dominated structure. The concept of ‘bending-dominated’ was first introduced by the Maxwell stability criterion [9]. The Maxwell stability criterion uses mathematical relationships to determine the structural stability of lattice structures. In a 2D lattice structure, the criterion is given by Equation (1),

(1) M=b−2j+3

In Equation (1), b refers to the number of struts, and j refers to the number of joints.

**Figure 1:**
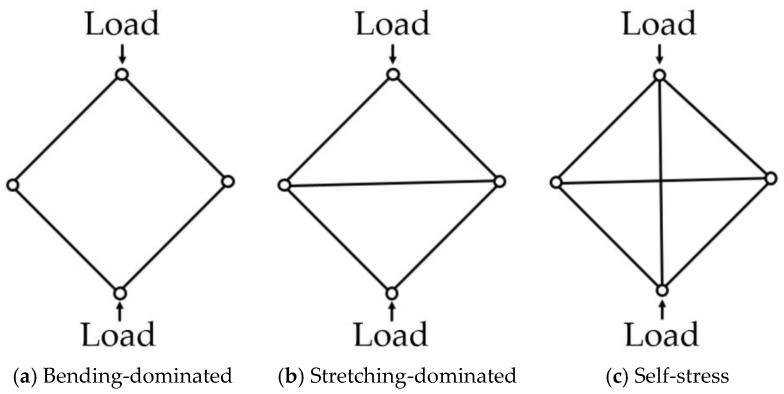
Three types of pin-jointed frames referred to in Maxwell stability theorem.

If M<0, bending behavior is dominant, and its structure is shown in Figure 1a. The lattice structure can be easily deformed under compressive loads because it has one or more degrees of freedom, but there exists no strength or stiffness in the direction of loading. In the case of M=0, the lattice structure exhibits stretching-dominated behavior, which is shown in Figure 1b. By adding auxiliary struts to a bending-dominated lattice structure, stretched struts reinforce the structure in the direction of loading and can make the lattice structure resist deformation at a higher strength. If M>0, stretching behavior is dominant, and this structure can be referred to as a self-stress structure. Self-stress refers to a state in which the combined forces of tension and compression within structural elements result in a net force of 0. It serves as a crucial indicator in assessing load-bearing capacity. When compressive load is applied on the structure, the vertical strut is shortened, and it pulls the struts jointly. Horizontal auxiliary struts are stretched and reinforced to better withstand tension forces [9,10].

**Figure 2:**
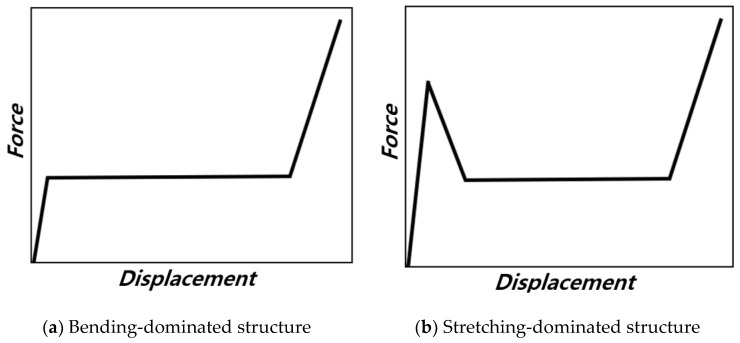
Schematic force–displacement curve for two types of lattice structure.

Moreover, whether a structure exhibits bending-dominated or stretching-dominated behavior can be determined through force analysis, based on the compressive displacement of the structure. Figure 2 illustrates the compressive force as a domain of compressive displacement for structures that are Figure 2a bending-dominated and for those where they are Figure 2b stretching-dominated, as indicated in the Maxwell stability criterion [11]. In the case of structures which are bending-dominated, even after passing through the elastic deformation region and reaching the plastic deformation region, the compressive force caused by deformation maintains a plateau value while absorbing energy. On the other hand, for structures which are stretching-dominated, the additional vertical or horizontal struts lead the structure to withstand higher loads in the elastic deformation region before reaching the plastic deformation region. Following plastic deformation, stress sharply decreases, and energy absorption proceeds with reduced load.

In this study, this idea is applied to the BCC structure because its 2D projection shape matches the configuration shown in Figure 1a. In previous studies, we can find work where auxiliary vertical struts were considered regarding the compressive behavior of lattice structures [5,6,8]. However, little attention has been given to horizontal struts in most previous works, despite their potential. Therefore, considerable attention has been given to horizontal struts for the first time in this work, since they may change the load-carrying mechanism and consequently withstand much higher loads, which can increase the energy absorption capability.

Although lattice structures vary depending on their internal arrangement, there are many difficulties in manufacturing and applying them in various fields due to the complexity of their shapes. This is because lattice structures are thin strut-based structures, and struts are easy to break during the process of drilling an internal hole in the manufacturing process. However, with the recent adoption of additive manufacturing technology, the fabrication of lattice structures has become easier, and related research is being conducted accordingly. Metal additive manufacturing technology, called metal 3D printing, is one of the outstanding additive manufacturing methods and shows different characteristics depending on the type of powder and manufacturing method [12,13,14,15]. Among the various metal additive manufacturing methods, SLM (Selective Laser Melting) and EBM (Electron Beam Melting) stand out as widely applied in many industries [16,17,18,19]. SLM uses a laser to melt the metallic powder and stack up to build a structure. EBM, on the other hand, uses an electron beam to melt the metallic powder and stack up like SLM. Utilizing these metal additive manufacturing methods revolutionized the fabrication of intricate lattice structures that are difficult to manufacture with general methods [20]. Indeed, both EBM and SLM have garnered considerable attention and widespread adoption in various industries, including dentistry and aerospace. Their applications have been driven by their distinctive capabilities and inherent advantages.

In this study, we considered relatively large size unit cell BCC lattice structures with horizontal structs. Therefore, we intended to manufacture the lattice structures without support, which should be eliminated after the 3D printing process. For this reason, we implemented the EBM method because it can produce a lattice structure with a large size unit cell without support compared to SLM.

Meanwhile, Ti-6Al-4V is renowned for its high strength, corrosion resistance, and heat resistance. Due to these advantages, it is commonly used in high-performance structures across aerospace, shipbuilding, automotive, and other industries. Because of these advantages, Ti-6Al-4V was chosen as a lattice structure material in this work.

A comprehensive investigation was conducted to figure out an effective unit lattice structure, using FEA to focus on the compressive mechanism and stress distribution of each strut. We reveal that the newly designed lattice structure offers significant advantages in structural robustness, with energy absorption increased by 365% compared to existing structures.

The newly designed lattice structures offer enhanced energy absorption and structural robustness, making them ideal for industries requiring lightweight and high mechanical performance structures. In aviation and navigation applications, they improve fuel efficiency and impact resistance, while in architecture, they enable strong, lightweight frameworks and improved safety. These advancements hold significant potential for transforming designs in sectors prioritizing strength, weight reduction, and resilience.

This paper is organized as follows: First, in Section 2 “Designing Lattice Structure”, novel lattice designs enhanced with auxiliary materials based on the Maxwell stability criterion are developed and compared against conventional BCC lattice structures to identify the most efficient energy-absorbing configuration. In Section 3, “Numerical Simulation”, we focus on numerical simulations, employing finite element analysis (FEA) to examine compressive behavior, stress distribution, and deformation mechanisms under compressive forces, providing critical insights for structural design. In Section 4 “Energy Absorption Indicators”, various performance indicators are analyzed to evaluate energy absorption metrics. Fabrication is addressed in Section 5, “Fabrication of Lattice Structure”, where lattice structures are produced using the EBM method, followed by quasi-static compression testing to obtain empirical data. Finally, Section 6, “Results and Discussion”, presents a thorough comparison of experimental results with FEA predictions, validating the reliability of the proposed lattice design and demonstrating its superior energy absorption performance [21,22,23].

## 2. Designing Lattice Structure

**Figure 3:**
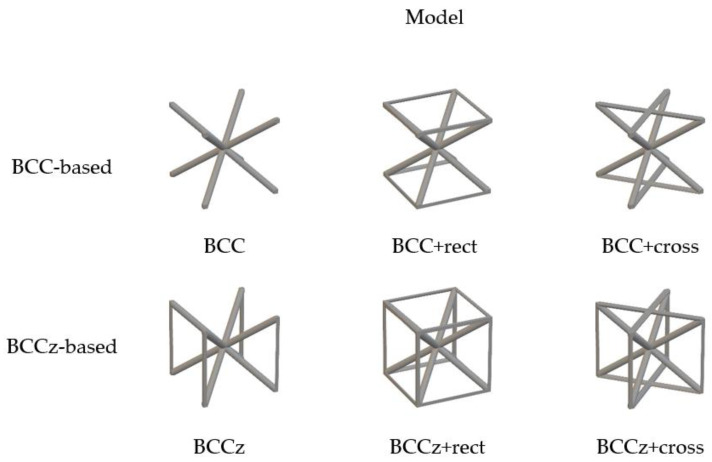
Computer-Aided Design of BCC-based structures and BCCz-based structure unit cells.

Auxiliary struts on a BCC are designed based on the Maxwell stability criterion. By adding horizontal and vertical struts on bending-dominated structures, it becomes stretching-dominated, as shown Figure 1. By adding vertical struts and horizontal struts on a BCC structure, it can be stiffer and withstand higher stress. And it allows for the increased energy absorption of the lattice structures. The auxiliary vertical strut is called the z strut, and the horizontal strut is divided into two types, rect and cross. If a BCC structure is reinforced with rectangular and cross-shaped horizontal struts, it is denominated as BCC+rect and BCC+cross, respectively. If vertical struts are added to a BCC structure, it is denominated as BCCz. If a BCC structure is reinforced with vertical and rectangular and cross-shaped horizontal struts, it is denominated as BCCz+rect and BCCz+cross, respectively. A total of six kinds of unit lattice structures are summarized in Figure 3. The unit cell size of the structure is 12 mm × 12 mm × 12 mm, and the thickness of each strut is a uniform 0.25 mm radius circle.

The design of a macrocellular lattice energy absorber leverages its lightweight and mechanically efficient characteristics, making it suitable for crashworthiness applications. The structure comprises periodic unit cells with an edge length of 12 mm and strut radius of 0.25 mm, resulting in a high void fraction that enhances energy absorption. The size effect, arising from the small strut radius relative to the unit cell size, significantly influences mechanical behavior, including surface-to-volume ratio effects, stress localization, and material gradients, which can alter yield strength and failure patterns [24]. The thin struts are prone to elastic or plastic buckling under compression, facilitating progressive collapse and energy dissipation through localized deformation. This interplay between geometry and the size effect enables the lattice to balance strength, stiffness, and energy absorption effectively, optimizing its performance for impact applications while maintaining minimal weight [25].

## 3. Numerical Simulation

**Figure 4:**
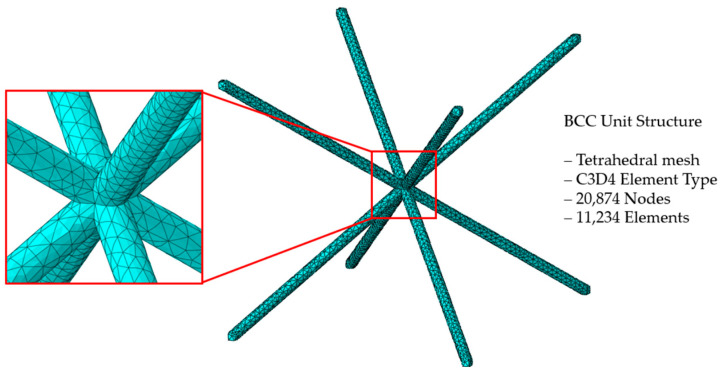
Illustration of finite element model of the BCC unit cell structure.

To predict and compare the energy absorption of designed structures, FEA was conducted. FEA is a computational method used to predict the behavior and response of structures by dividing the structure into a finite number of elements and calculating their interactions based on physical principles. It presents lots of advantages in reducing experimental cost and understanding the compressive deformation mechanism while absorbing energy. The commercial software ABAQUS/explicit 2022 was used to model the numerical simulation of quasi-static compression analysis [26]. To ensure a more precise assessment of stress distribution and the compressive behavior of the unit structure, quasi-static compression analysis was performed on the unit structure composed of solid elements (C3D4). The number of nodes was 20,874, and the number of elements was 11,234 in the BCC unit structure. The element detail of the BCC unit structure is depicted in Figure 4, and the element details of other unit lattice structures are provided in Appendix A.1.

**Figure 5:**
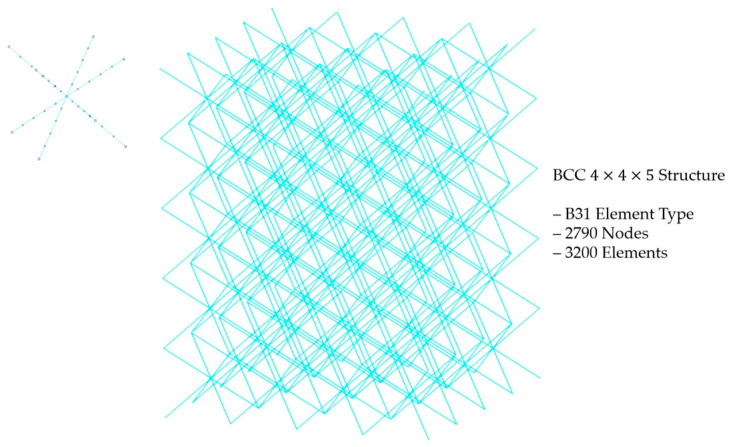
Illustration of a finite element model of a BCC 4 × 4 × 5 lattice structure.

After analyzing the compressive behavior and stress distribution, quasi-static compressive analysis of 4 × 4 × 5 lattice structure was conducted. Considering the substantial number of elements present in the 4 × 4 × 5 lattice structure, the division into solid elements using an explicit method would be time-consuming and computationally intensive. Therefore, we opted for a more efficient approach by dividing the structure into beam elements, which provides simplified models. Although beam element offers a less detailed representation of the lattice structure compared to solid elements, they significantly reduce computational time and resource requirements, while still allowing us to gain valuable insights into the overall behavior and performance of the lattice structure during quasi-static compression. A 4 × 4 × 5 BCC lattice structure composed of beam element (B31) with 2790 nodes and 3200 elements was investigated through quasi-static compression analysis [27]. The element detail of the BCC 4 × 4 × 5 lattice structure is depicted in Figure 5, and for other variants of the BCC structure, detailed information is included in Appendix A.2 of the revised paper.

The calibrated mechanical properties of Ti-6Al-4V, including its plastic behavior, are applied to the structure [28,29]. In the FEA analysis, two rigid plates are positioned, one beneath and one above the lattice structure. The top plate compresses the structure at a uniform velocity of 12 mm/s for unit structures and 60 mm/s for 4 × 4 × 5 lattice structures, with uniaxial motion, while the bottom plate remains fixed. During compression analysis, plates and structures are under a contact condition with an impenetrability condition on normal behavior and a friction coefficient of 0.3 on tangential behavior. The boundary conditions of quasi-static under compressive loading analysis are depicted in Appendix A.3 and Appendix A.4.

**Figure 6:**
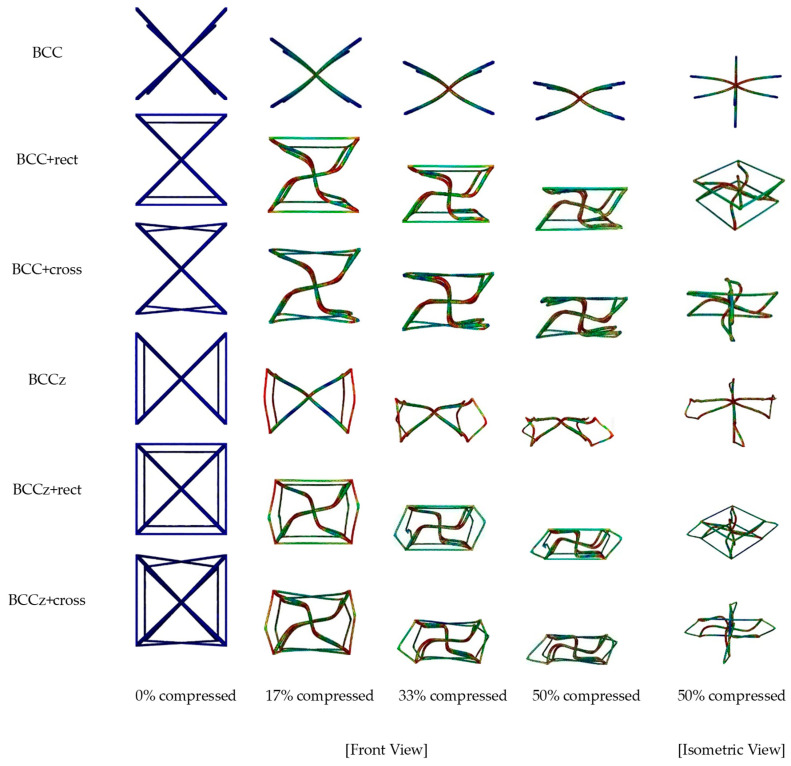
Deformation mechanism of unit structures in FEA quasi-static compression analysis.

Figure 6 exhibits quasi-static compressive behaviors under compressive displacement of six types of unit structures. The BCC unit structure showed a pattern where the angle of the crossed struts widened during the compression process due to the concentration of stress at the intersection of each strut during compression. On the other hand, in the case of structures with horizontal struts, relatively high stress occurs on the horizontal struts, not at the intersection of the BCC-part struts. Also, the angle of the intersection of the BCC part is maintained, being rotated by the BCC-part strut during compression.

When vertical struts are added on a BCC structure, high stress occurs in both the vertical auxiliary elements and the intersection of struts during compression. And its compressive behavior resembles the BCC structure. Furthermore, when both vertical and horizontal struts are added, the influence of the horizontal auxiliary struts leads to high stress at the intersections of the horizontal and vertical auxiliary struts and the middle part of the BCC-part struts. Similar to the previous case, the BCC part exhibits a behavior where it rotates in a pinwheel-like motion when compressed.

**Figure 7:**
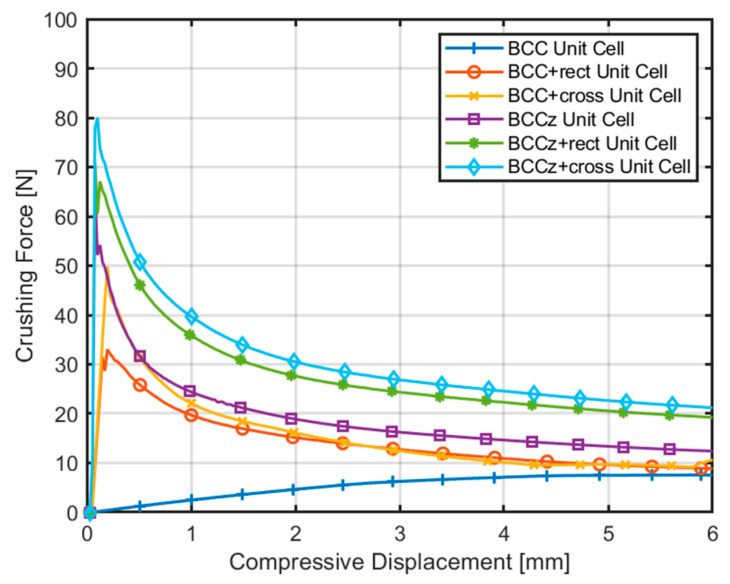
Comparison of compressive displacement–crushing force curves of unit structure.

The compressive displacement–crushing force curves derived from quasi-static compression analysis on the unit structure are shown in Figure 7. According to the graph, the BCCz+cross withstands the highest initial maximum force, followed by BCCz+rect, BCCz, and BCC+cross. And it also has the highest plateau force when compressed up to 6 mm, followed by BCCz+rect, BCCz, and BCC+cross. Through this, it was confirmed that the vertical auxiliary struts and horizontal auxiliary struts, especially cross, contribute much to the initial maximum load and plateau force. Also, the compressive displacement–crushing force curves of the BCC structure show a pattern of bending-dominant behavior, and as for the structure with either vertical or horizontal struts added on the BCC structure, compressive displacement–crushing force curves with stretching-dominant behavior are observed [11].

**Figure 8:**
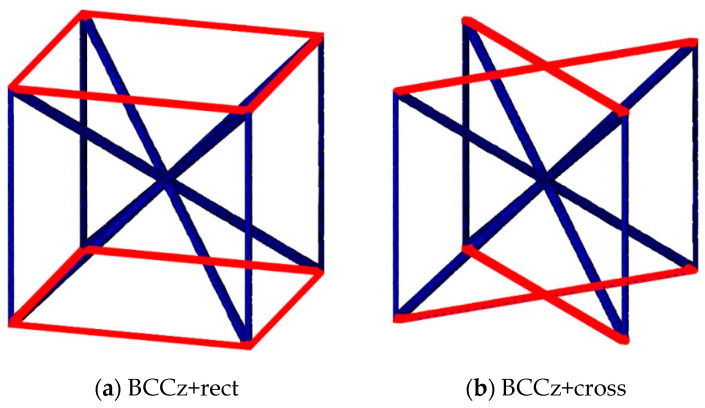
Highlighted elements of horizontal auxiliary struts of the structures.

To examine the energy absorbing contribution between the two types of horizontal auxiliary struts, which exhibit similar compressive behavior, the average stress occurring on the entire elements of the cross horizontal auxiliary elements and rectangular horizontal auxiliary elements was examined and compared. As shown in Figure 8, the elements of horizontal auxiliary struts are highlighted in red. Comparison of mean stress was conducted between BCC+rect and BCC+cross, as well as between BCCz+rect and BCCz+cross.

**Figure 9:**
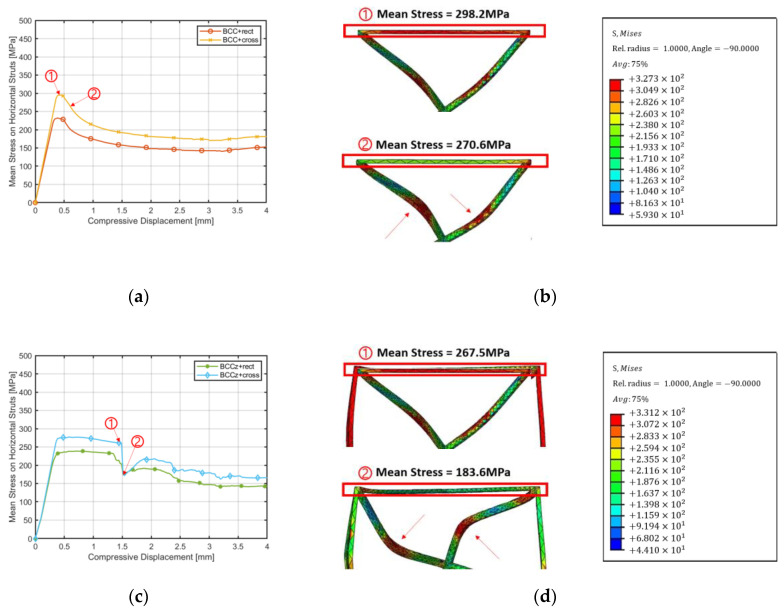
Comparison of mean stress under compressive displacement. (**a**) Displacement–Mean Stress Curve of BCC-based Structures. (**b**) Mean Stress of Horizontal Auxiliary Struts of BCC-Based Structures. (**c**) Displacement–Mean Stress Curve of BCCz-based Structures. (**d**) Mean Stress of Horizontal Auxiliary Struts of BCCz-Based Structures.

Figure 9 illustrates a comparison of mean stress under compressive displacement curves for the elements of horizontal auxiliary struts in Figure 9a, a BCC-based structure, and Figure 9c, a BCCz-based structure, with the addition of their respective horizontal auxiliary struts. It was observed that in both BCC-based and BCCz-based structures, the average stress generated in the elements of cross auxiliary struts during compression was higher than elements of rectangular auxiliary struts. The reason for higher stress in the horizontal auxiliaries during compression at the same displacement is akin to principles seen in tensegrity structures, where increased tension in the auxiliaries is necessary for the deformation of the structure, allowing it to withstand higher loads [30,31].

Figure 9b exhibits snapshots of structural behavior concerning the average stress in horizontal auxiliary struts. In the BCC-based structure, initial compression leads to high-stress concentrations within the horizontal auxiliary struts. However, as the central part BCC strut starts to bend during compression, stress levels in the horizontal auxiliary struts experience a reduction. The presence of horizontal auxiliary struts restrains the widening of the BCC-part struts, causing high stress generation during this phase. Notably, the compression behavior transforms from a BCC-part strut expansion to a bent configuration at the center of the BCC-part strut, leading to reduced stress levels after reaching the peak stress within the horizontal auxiliary struts.

In Figure 9d, for the BCCz-based structure with horizontal auxiliary struts, the stress distribution reveals similar patterns. Initially, during elastic deformation of the horizontal auxiliary struts, high stress is evident, followed by a sharp decline in average stress. Importantly, this high-stress manifestation extends to the connected horizontal auxiliary struts. With further compression, the vertical auxiliary struts transition from elastic to plastic deformation. During this transition, stress levels in both the vertical and connected horizontal auxiliary struts undergo a reduction.

**Figure 10:**
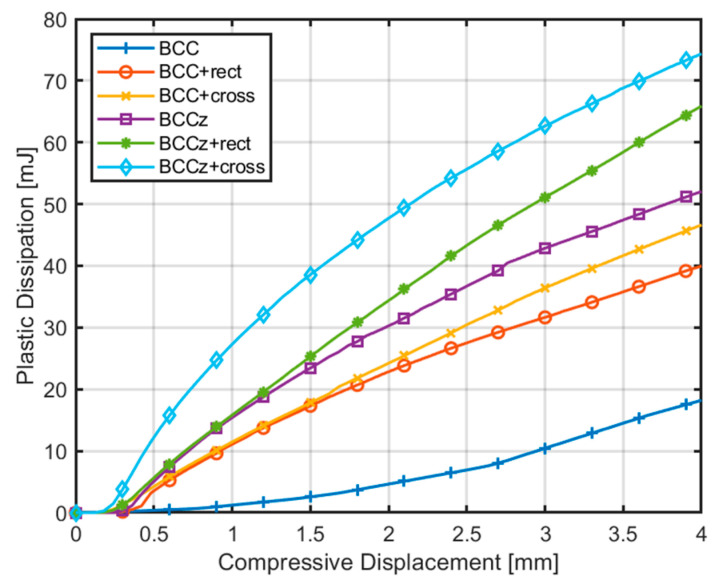
Comparison of plastic dissipation of unit structure.

An analysis to examine the implication of plastic deformation resulting from structural fracture as a primary factor contributing to changes in internal energy was conducted [32,33]. Internal energy, $E_{I},$ can be expressed as the sum of elastic strain energy, $E_{S},$ and energy dissipated by plasticity, $E_{p},$ as shown in Equation (2). Therefore, we focused on quantifying the dissipated energy, i.e., the energy absorption, concerning the compressive displacement of the unit structure. The energy dissipated by plastic deformation curve is exhibited in Figure 10. Structures encompassing both vertical and horizontal auxiliary struts exhibited the highest energy absorption capacity. Additionally, it was observed that structures with vertical auxiliary struts outperformed those with horizontal auxiliary struts alone in terms of energy absorption. Furthermore, among horizontal auxiliary struts, it was evident that cross-shaped horizontal auxiliary struts outperformed rectangular auxiliary struts in terms of energy absorption.

(2) $$\begin{array}{c} E_{I}=\int_{0}^{t}(\int_{V}\sigma^{c}:\dot{\varepsilon }dV)d\tau \\ =\int_{0}^{t}(\int_{V}\sigma^{c}:{\dot{\varepsilon }}^{el}dV)d\tau +\int_{0}^{t}(\int_{V}\sigma^{c}:{\dot{\varepsilon }}^{pl}dV)d\tau \\ =E_{S}+E_{P} \end{array}$$

**Figure 11:**
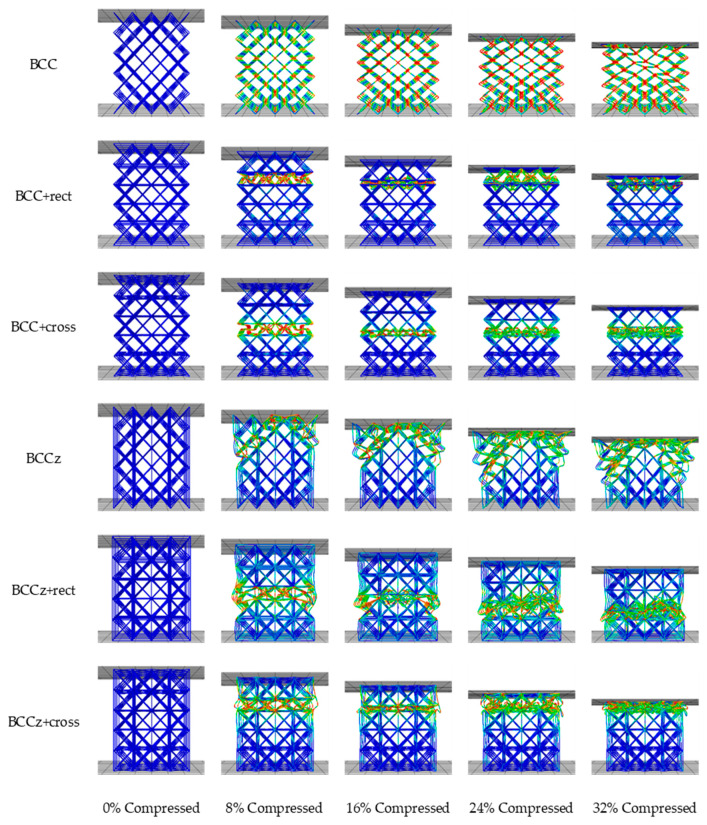
Deformation mechanism of six types of 4 × 4 × 5 structures in FEA quasi-static compression.

After confirming the deformation mechanism of the unit structure, a quasi-static compression analysis was conducted on a structure consisting of multiple unit structures. Each unit structure had dimensions of 12 mm × 12 mm × 12 mm, with a 0.25 mm radius thickness. The structure had dimensions of 48 mm × 48 mm × 60 mm and contained a 4 × 4 × 5 number of unit structures, for a total of 80. The analysis was performed using the ABAQUS/explicit method, the same as unit structure. A quasi-static compression analysis was carried out with an upper rigid plate with a compressive velocity of 60 mm/sec downward, and contact conditions were the same as those used in the unit structure analysis. In Figure 11, the deformation mechanisms of a 4 × 4 × 5 structure are exhibited.

**Figure 12:**
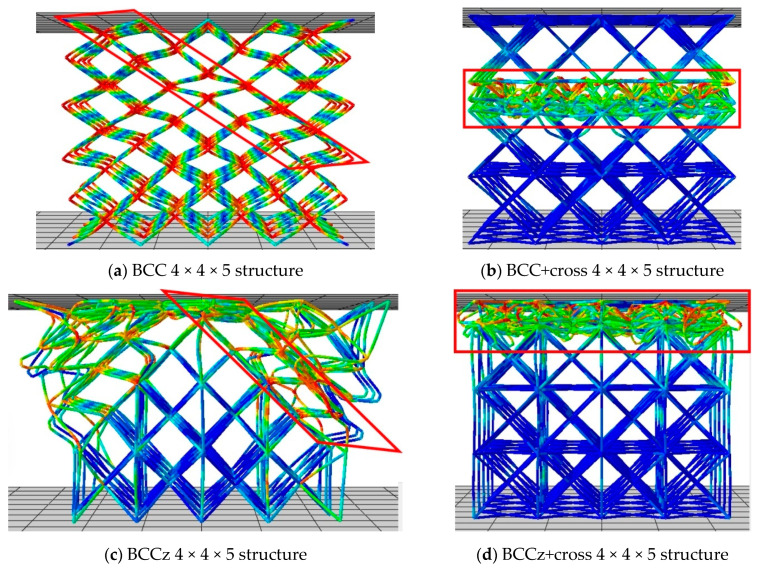
Deformation aspects of 32% compressed BCC-based and BCCz-based structures.

Figure 12 exhibits the compressive mechanism of 4 × 4 × 5 BCC and BCC+cross structures. In the case of the BCC structure, as the compression proceeded, high stress occurred on the cross-section of struts, resulting in widening the struts and continued compression behavior similar to the behavior observed in the unit structure analysis. This led to overall structural deformation occurring in the diagonal direction fracture. However, the BCC+cross structure exhibited different compressive behavior. As the compression proceeded, high stress occurred on the middle of BCC-part struts, instead of at the intersections of the struts. Consequently, structural deformation triggered a layer-by-layer fracture. 

The compressive mechanism of the structures when vertical struts were added on BCC and BCC+cross structures is exhibited in Figure 12a–d. The compressive mechanisms of the structures are similar. If horizontal auxiliary struts are not added, diagonal fracture occurs. On the other hand, a layer-by-layer fracture occurs when horizontal auxiliary struts are added.

This demonstrates that the addition of horizontal auxiliary parts can significantly alter the behavior and performance of the lattice structure during compression. And addition of vertical auxiliary parts increases the energy absorption without altering its compressive mechanism. The redistribution of stress and the rotation or fracture of partial struts indicate improved load-bearing capacity and energy absorption capabilities in the presence of auxiliaries.

In conclusion, it was observed that when the central angle of the struts in the BCC part is not maintained during compression, the overall structure becomes vulnerable to diagonal collapse. Conversely, when the central angle of the struts in the BCC part is maintained, the entire structure becomes vulnerable to gradual layer-by-layer collapse.

To identify the vulnerable stress mechanisms of the entire lattice structure, specific areas where layer-by-layer or diagonal deformation occurred during the compression were extracted. Boundary conditions were applied to these areas to induce the intended deformation, and the force at the point of structural deformation was recorded. This allowed us to determine the stress mechanisms to which the structure is most vulnerable.

**Figure 13:**
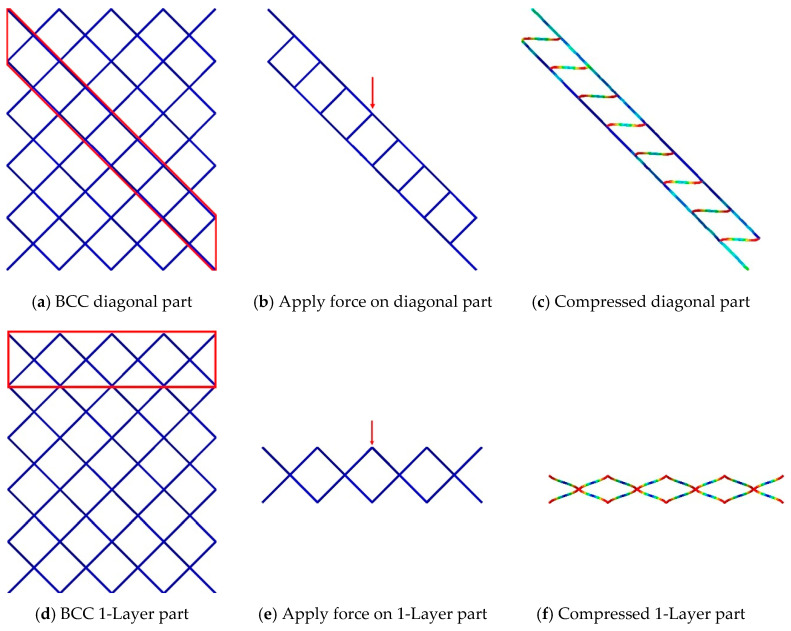
Extracting diagonal part and 1-Layer part of BCC structure.

When diagonal collapse occurs in the BCC 4 × 4 × 5 structure, only the portion where deformation occurs was extracted, as shown in Figure 13a. The boundary condition of compression with a velocity of 12 mm/s downward induces diagonal fracture. Figure 13b and c indicate a diagonally deformed structure, which is similar to the deformed motion of the BCC 4 × 4 × 5 structure in Figure 12. Similarly, in Figure 13d, only the portion where one layer of damage could occur in the BCC 4 × 4 × 5 structure was extracted, as shown in Figure 13e, and a boundary condition of compression with a velocity of 12 mm/s was applied in the vertical direction. It was observed that damage occurred layer-by-layer, as shown in Figure 13f.

**Figure 14:**
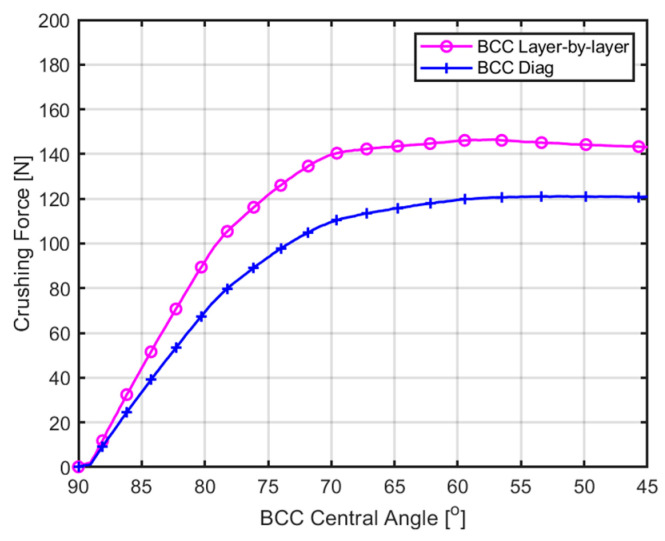
BCC central angle–crushing force curve for 1-Layer part and diagonal part of BCC.

Figure 14 illustrates the forces required for diagonal and layer-by-layer collapse in the BCC structure. Here, the ‘BCC Central Angle’ on the *X*-axis represents the angle formed by connecting the center point of the BCC with points on the upper and lower portions of the structure. This angle is used to indicate the force generated during the same compression displacement in the BCC part. In the case of diagonal collapse, as the force increases, the deformation of the structure occurs gradually, resulting in a gradual decrease in the angle of the BCC central part. Similarly, for layer-by-layer collapse, it can be observed that crushing force increases gradually as the angle of the BCC central part decreases. Furthermore, for structures experiencing diagonal collapse, yield occurs at approximately 98 N, and as deformation progresses in the BCC section, the change in force required for further deformation becomes relatively modest. In structures undergoing layer-by-layer collapse, compressive yield occurs at around 120 N. These results indicate that the BCC 4 × 4 × 5 structure requires relatively lower force for diagonal collapse than layer-by-layer, suggesting a significant propensity for diagonal damage to occur during compression.

**Figure 15:**
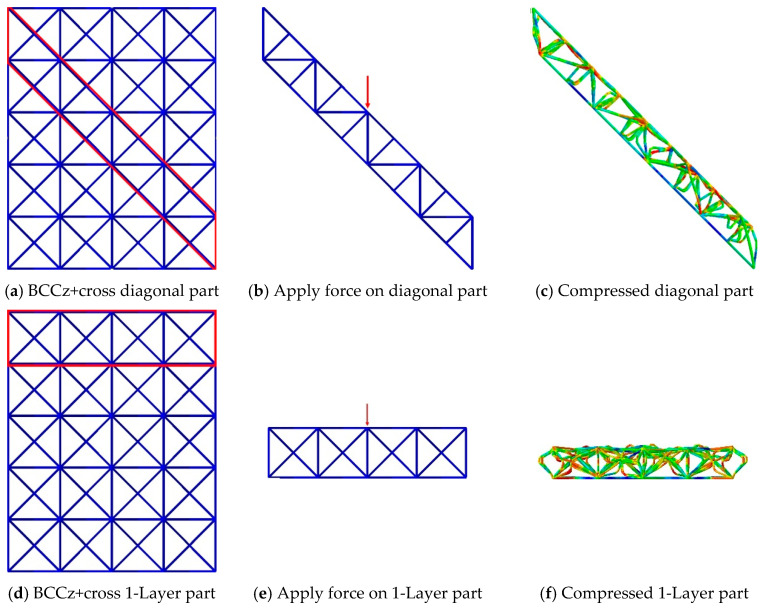
Extracting diagonal part and 1-Layer part of BCCz+cross structure.

Figure 15 illustrates the analysis conducted on the BCCz+cross 4 × 4 × 5 structure, following a similar approach to the previous BCC structure. Figure 15b is designed to induce diagonal damage, and Figure 15e represents progressive layer-by-layer collapse, resembling actual behavior. A key difference in compression behavior between the diagonal part of BCC structure and the diagonal part of BCCz+cross structure is the existence of the struts, which directly support the vertical compression. For the BCC structure, when compressive deformation is applied on a diagonal part, it simply folds, which promotes a bending-dominated structure. On the other hand, for the BCCz+cross structure, when compressive deformation is applied, the presence of vertical and horizontal auxiliary struts opposes the struts so that they cannot be compressed easily. And it promotes stretching-dominated behavior, like the Maxwell stability criterion.

**Figure 16:**
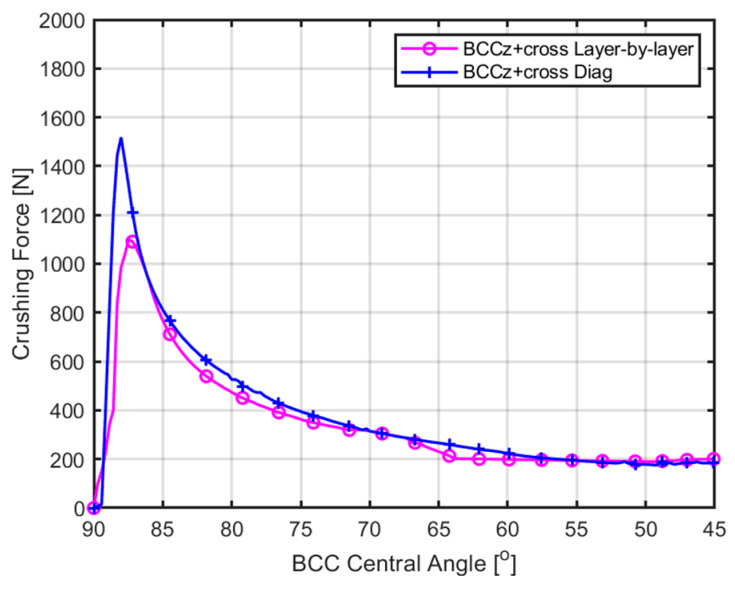
BCC central angle–crushing force curve for 1-Layer part and diagonal part of BCCz+cross.

Figure 16 represents a graph illustrating the force applied during diagonal damage and layer-by-layer collapse in the BCCz+cross structure. In the case of diagonal damage in the BCCz+cross structure, compressive yield occurred after reaching a maximum force of approximately 1550 N. For the layer-by-layer collapse mechanism in the BCCz+cross structure, compressive yield was observed after reaching a maximum force of about 1123 N. Consequently, this indicates that the BCCz+cross structure is more susceptible to compression stress during layer-by-layer collapse than diagonal collapse.

## 4. Energy Absorption Indicators

Energy absorption indicators presented in this study are used to evaluate the energy absorbing performance of the structures and can be calculated from the raw data of compression analysis. And indicators make it easier to compare the energy absorbing abilities of the structures quantitatively. These indicators can be summarized as follows [34,35,36]:

### 4.1. EA, Energy Absorption

(3) $$EA=\int_{d_{start}}^{d_{end}}F\;dx$$

**Figure 17:**
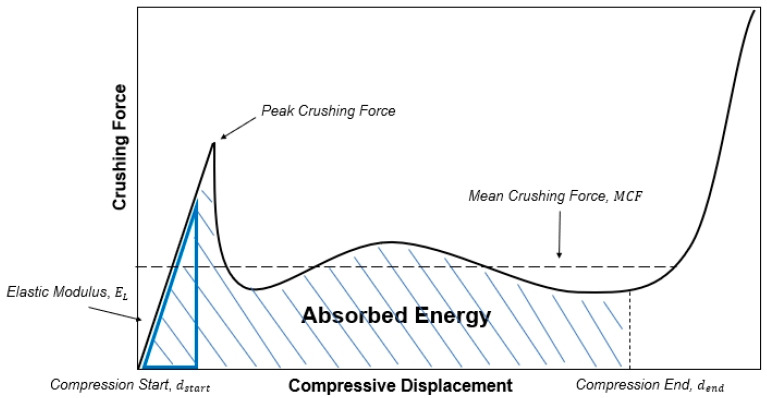
Schematic of typical displacement–force curve of lattice structure under quasi-static compression.

EA in Equation (3) quantifies the total amount of energy that the structure can absorb during the process of compression and plastic deformation induced by the impact. F refers to the crushing force of lattice structure, $d_{start}$ refers to the displacement when compression starts, $d_{end}$ refers to the displacement when lattice structure is fully densified. By evaluating EA, one can ascertain the capacity of the structure to dissipate and absorb energy, which is crucial for mitigating the potential damage caused by impact events. A typical displacement–force graph of energy-absorbing tests through plastic deformation of energy absorbing structures is shown in Figure 17, and its area of graph can be expressed as absorbed energy.

### 4.2. SEA, Specific Energy Absorption

(4) $$SEA=\frac{1}{m}\;\int_{d_{start}}^{d_{end}}F\;dx$$

SEA in Equation (4) represents the amount of energy absorbed by a structure per unit mass. It provides a means of comparing the energy absorption performance of structures with varying masses. m refers to the mass of the lattice structure. In general, an increase in the number of struts results in higher energy absorption due to the corresponding increase in mass. By examining the SEA value, efficiency of energy absorption relative to the mass of the structure can be assessed, enabling comparisons between different structural designs in terms of their impact resistance capabilities.

### 4.3. MCF, Mean Crushing Force

(5) $$MCF=\frac{1}{d_{end}-d_{start}}\int_{d_{start}}^{d_{end}}F\;dx$$

MCF in Equation (5) represents the average compressive load at which a structure undergoes plastic deformation during impact. When subjected to an impact force, the structure experiences corresponding compressive forces. If the compressive load exceeds the impact force, the structure remains intact without absorbing the shock. Conversely, if the compressive load is insufficient compared to the impact force, the structure sustains damage before it can effectively absorb the impact, rendering it incapable of shock absorption. Hence, MCF serves as an indicator to determine the suitability of a shock-absorbing structure based on the specific impact scenario.

### 4.4. STD, Standard Deviation of Crushing Force

(6) $$STD=\frac{1}{MCF}\sqrt{\frac{\int_{d_{start}}^{d_{end}}{\left|F-MCF\right|}^{2}\;dx}{d_{end}-d_{start}}}$$

STD in Equation (6) is calculated by dividing the standard deviation of compressive load by MCF. Generally, it is desirable to have low variability in the compressive load. Excessive variability in the compressive load can result in significant vibration of the subject experiencing the impact, posing a safety risk. Therefore, STD is utilized as a safety indicator for assessing the impact resistance of a structure. By evaluating the STD value, one can gauge the variability and consistency of the crushing force during energy absorption, thereby ensuring the effectiveness and reliability of the structure in absorbing energy.

### 4.5. SMCF, Specific Mean Crushing Force

(7) $$SMCF=\frac{1}{d_{end}-d_{start}}\;\frac{1}{m}\;\int_{d_{start}}^{d_{end}}F\;dx$$

SMCF in Equation (7) represents the amount of energy absorbed per unit length and unit mass. It is computed by dividing EA by the mass of the structure and the displacement at which energy is absorbed. In the energy-absorbing structures in aerospace, there are constraints on the mass and volume of the structure that can be accommodated on an aircraft. These constraints vary depending on the aircraft’s weight and lift. Therefore, SMCF serves as a criterion for selecting a structure that is appropriate in terms of mass and volume for a given situation. Generally, a higher SMCF value indicates that more energy is absorbed for the same level of compression under the same mass. This allows for a more quantitative assessment of energy absorption capabilities.

**Figure 18:**
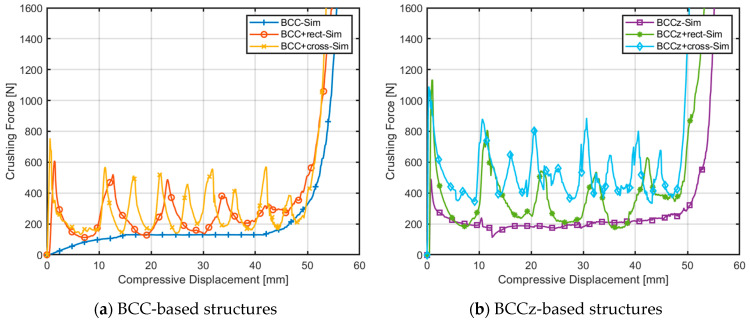
Comparison of compressive displacement–crushing force curves of FEA.

The compressive displacement–crushing force curves obtained from the finite element quasi-static compression analysis are compared in Figure 18. Energy absorption indicators of structures are compared in Table 1. In Figure 18, the crushing force of a BCC structure is gradually increased and maintains its plateau force. The crushing force of BCCz is a bit higher than BCC, and it also maintains its plateau force. If horizontal auxiliary struts are added on the structures, their crushing force fluctuates with increasing STD. Structures with cross horizontal auxiliary struts have a higher EA, SEA, MCF and SMCF than structures with rectangular auxiliary struts with a lower STD. Therefore, it is observed that cross horizontal auxiliary struts increase energy absorbing abilities more effectively than rectangular auxiliary struts.

The addition of auxiliary materials to the lattice structures resulted in significant improvements in the energy absorption performance. Here are the summarized findings:

1. BCC+cross structure: the addition of a cross horizontal auxiliary struts to the BCC structure increased EA by 265% and SMCF by 170%.
2. BCCz structure: the addition of vertical auxiliary struts to the BCC structure led to a 198% increase in EA and 160% increase in SMCF.
3. BCCz+cross structure: by adding both cross horizontal and vertical auxiliaries, the structure exhibited a substantial enhancement in energy absorption performance, with EA increasing by 369% and SMCF increasing by 275% compared to the BCC structure.

## 5. Fabrication of Lattice Structure

Based on the results from FEA, the autonomously designed BCCz+cross lattice structure demonstrated superior energy absorbing performance compared to other variations of the BCC lattice structure. Subsequently, the structure was fabricated using a metal 3D printing manufacturing technique known as EBM. In this technique, metal powder is exposed to electron beams, leading to the sintering and layer-by-layer printing of the metal powder to create the desired structure.

EBM offers distinct advantages over the commonly used SLM (Selective Laser Melting) method for lattice structure fabrication. It involves manufacturing the structure in a vacuum chamber, resulting in reduced residual stress within the fabricated product and mitigating the risk of hydrogen embrittlement-induced property degradation. Moreover, the high-temperature manufacturing process at approximately 700 °C minimizes product warping [37], and within the realm of metal additive manufacturing technology, the necessity for supports depends on the spacing between printed components and the stacking angle of these components. Notably, when employing the EBM method, fine powder serves as an intermediary support material between prints, and it can be readily removed. Although the surface finish may not be as refined, it is deemed acceptable for energy absorbing applications, making the EBM method a suitable choice [38].

**Figure 19:**
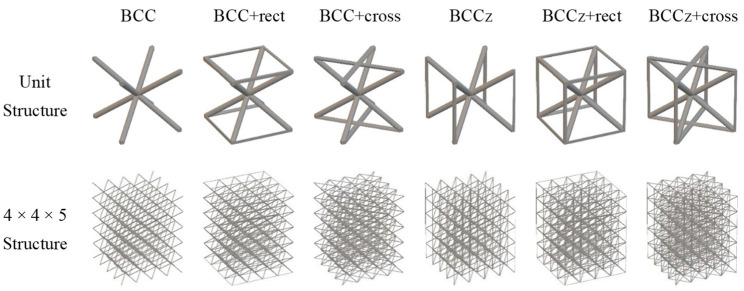
Configuration of unit structures and 4 × 4 × 5 structures.

For the fabrication process, six structures were output, each with a length of one corner of the unit structure set to 12 mm, consistent with the dimensions used in the FEA models. The overall size of the fabricated structure was 48 mm × 48 mm × 60 mm, with the number of unit structures in each corner set to 4, 4, and 5, respectively, as illustrated in Figure 19.

**Figure 20:**
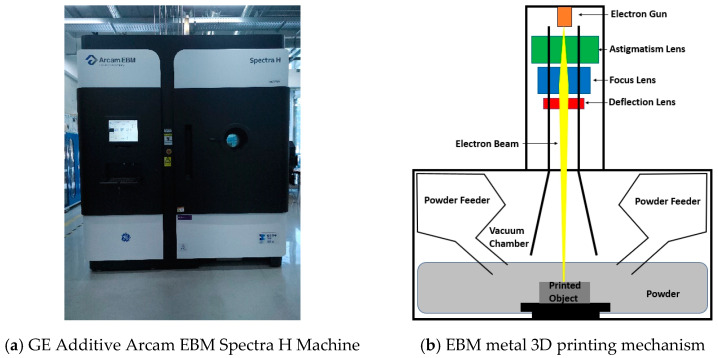
Electron Beam Melting 3D printer.

**Figure 21:**
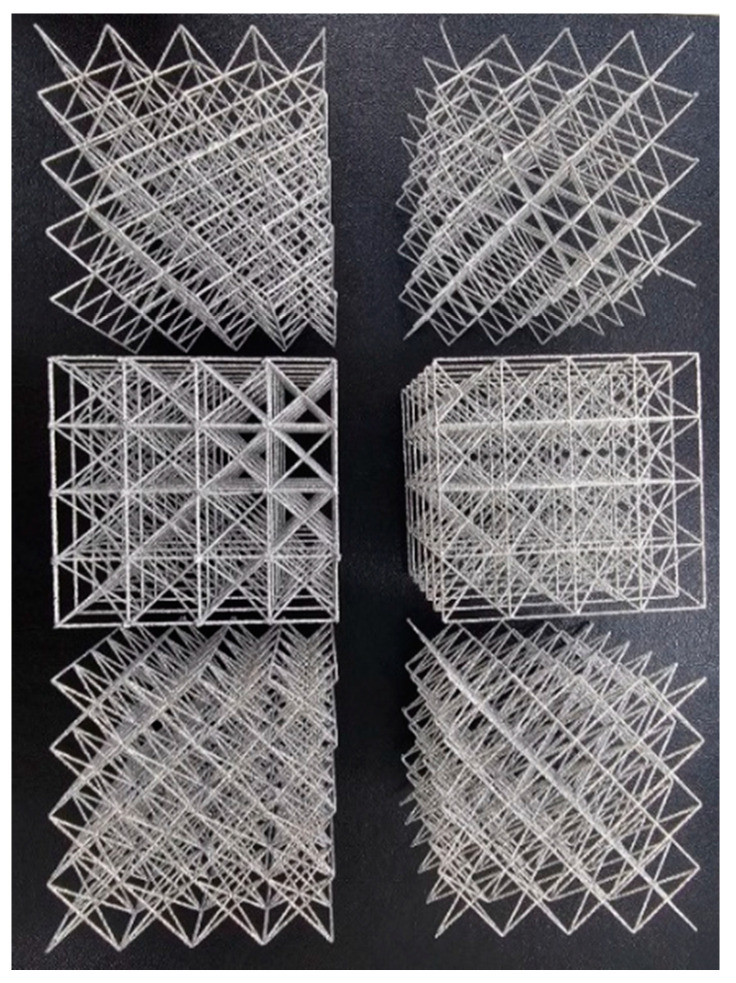
Metal 3D printed lattice structures.

**Figure 22:**
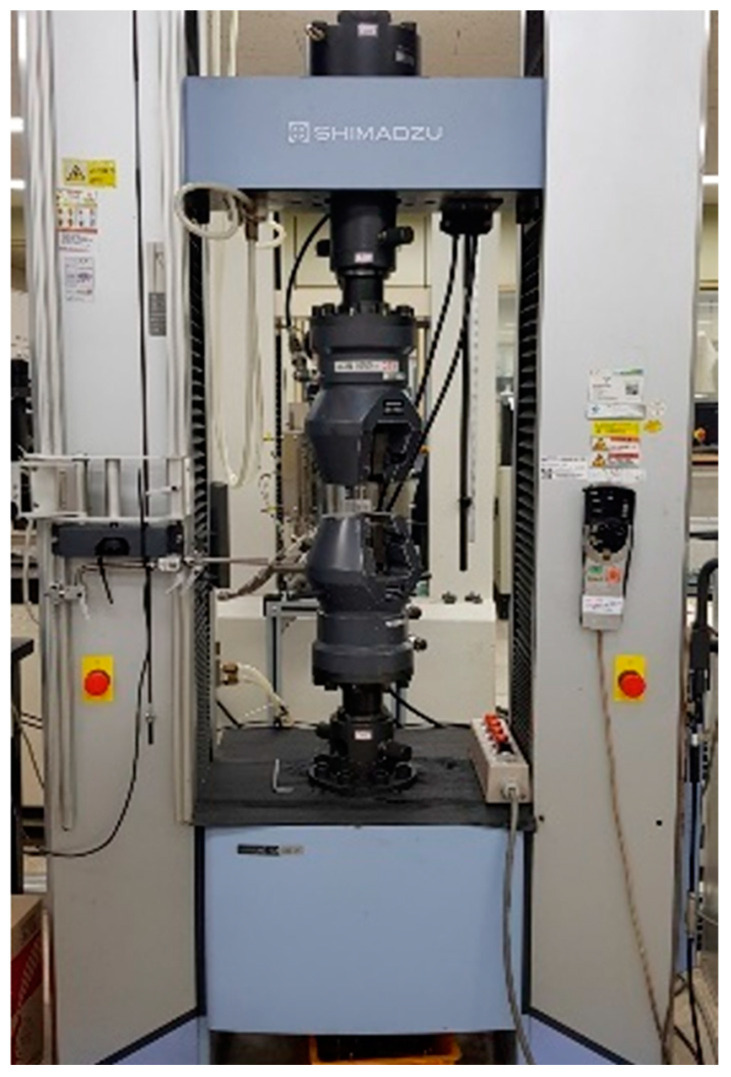
SHIMADZU UTM (Universal Testing Machine) AG-X Plus 300 kN.

The structures were manufactured by the EBM method with the GE Additive’s Arcam EBM Spectra H Machine, shown in Figure 20a. The fabricating process is illustrated in Figure 20b. The electron beam from an electron gun is regulated by the lens, and the focused laser beam melts the powder in vacuum chamber. Melted powder is stacked and fabricates the structure. Ti-6Al-4V Grade 5 metal powder was employed, possessing an average particle size ranging from 45 to 106 μm. During the fabrication process, the sintered powder was incrementally layered, with a thickness of 50 μm per layer. These parameters and machine settings were selected to ensure the successful production of the lattice structures using EBM. Fabricated structures are shown in Figure 21. For the structures, compressive tests were performed using the SHIMADZU universal testing machine, as shown in Figure 22.

## 6. Results and Discussion

**Figure 23:**
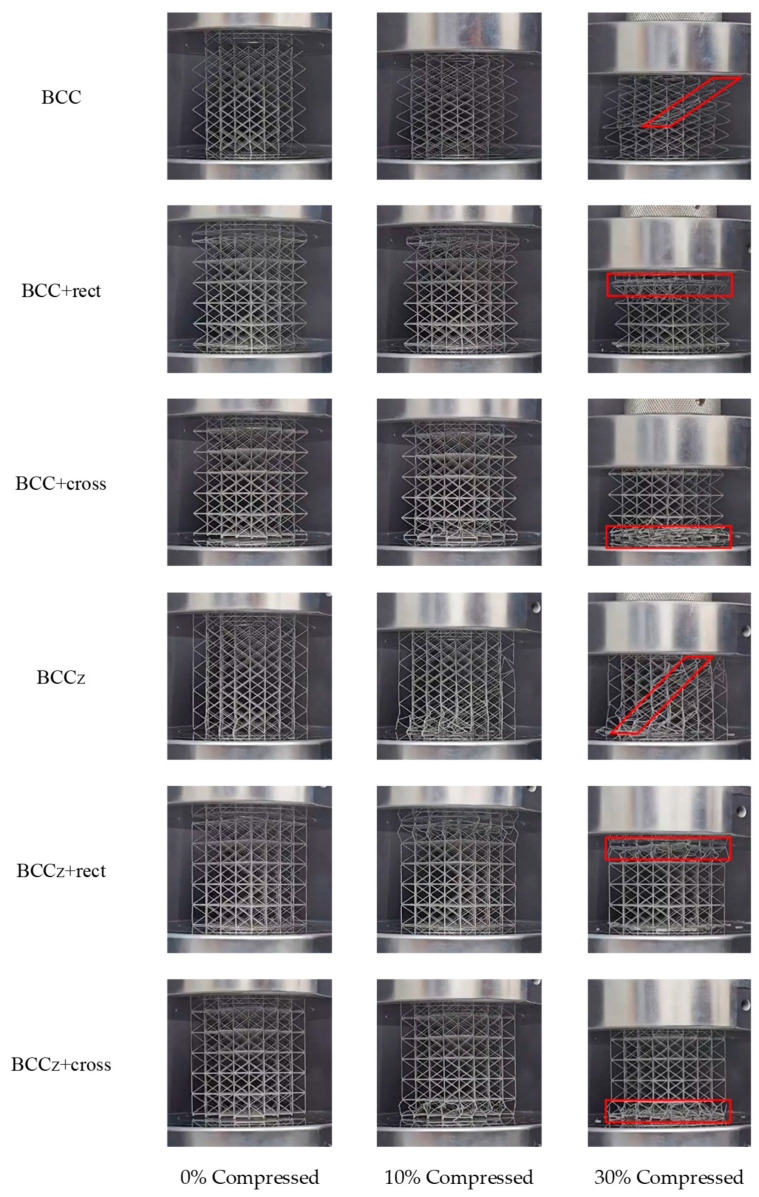
Compression behavior of EBM metal 3D printed structures.

Figure 23 depicts the compression behaviors of all structures. The BCC structure and BCCz structure exhibit overall collapse with diagonal fracture, whereas the structures with horizontal aids demonstrate a layer-by-layer collapse mechanism. It is observed that the BCC and BCCz unit structures undergo widening of the BCC part. In contrast, the unit structure with horizontal auxiliaries effectively inhibits this widening and assumes a pinwheel shape around the center of the BCC part. This distinction, as illustrated in Figure 6, demonstrates how the alteration in the unit structure contributes to the mechanism of the overall compressive modes, namely collapsing as a whole and collapsing layer-by-layer.

**Figure 24:**
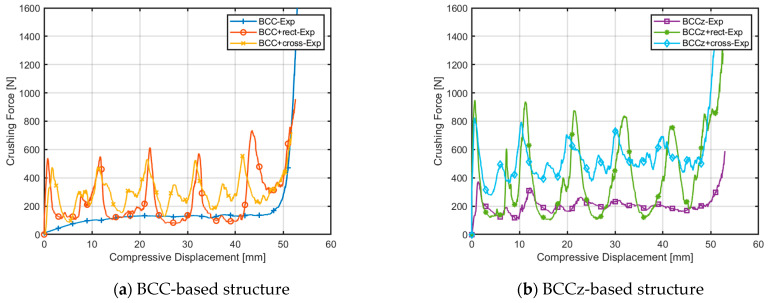
Comparison of compressive displacement–crushing force curves of EBM printed structures.

Figure 24 displays the plotted raw data obtained from the quasi-static compression experiments. The BCC structure exhibits a bending-dominant behavior, while the structures with added horizontal and vertical aids exhibit a stretching-dominant behavior. It was observed that the crushing force of the BCC structure increases smoothly and maintains its plateau crushing force, while for BCC with auxiliary struts, after reaching a peak strength, the structures experience compression due to plastic deformation.

In the case of the BCC structure, the entire structure undergoes compression, with the entire structure bearing the compressive load and maintaining it throughout the compression process. However, in the case of a structure with horizontal auxiliary struts added, the collapse occurs layer by layer. As each layer collapses, the next layer follows, resulting in a sequential collapse pattern. This leads to an increase in the compressive load until failure occurs, followed by a decrease in the compressive load. This cyclic process of increasing and decreasing compressive load repeats until the compressive displacement reaches densification [39].

And in Table 2, the BCC+cross resulted in a significant reduction in the STD value compared to the BCC+rect. Specifically, the STD decreased from 0.6848 to 0.3371 when the cross-shaped aids were utilized. This implies that the cross-shaped aids contribute to a more stable and consistent compressive load during the quasi-static compression test, leading to a decrease in variability and better control of the impact forces experienced by the structure. The MCF of BCC+cross is 2.00 × 10^3^, about 20% higher than that of BCC+rect, which has an MCF of 1.73 × 10^3^. As for BCCz, a similar mechanism arises that BCCz+cross has a higher MCF and lower STD than BCCz+rect.

Conversely, the incorporation of horizontal auxiliaries led to an enhancement in energy absorption performance, with a significant reduction in STD performance. Notably, the rectangular horizontal auxiliaries manifested a pronounced decline in STD performance. Experimental findings indicate substantial fluctuations in the crushing force when horizontal auxiliaries are introduced. These fluctuations can be attributed to a layer-by-layer occurrence of buckling and are exacerbated by the inherent brittleness of the thin metal struts [40].

**Figure 25:**
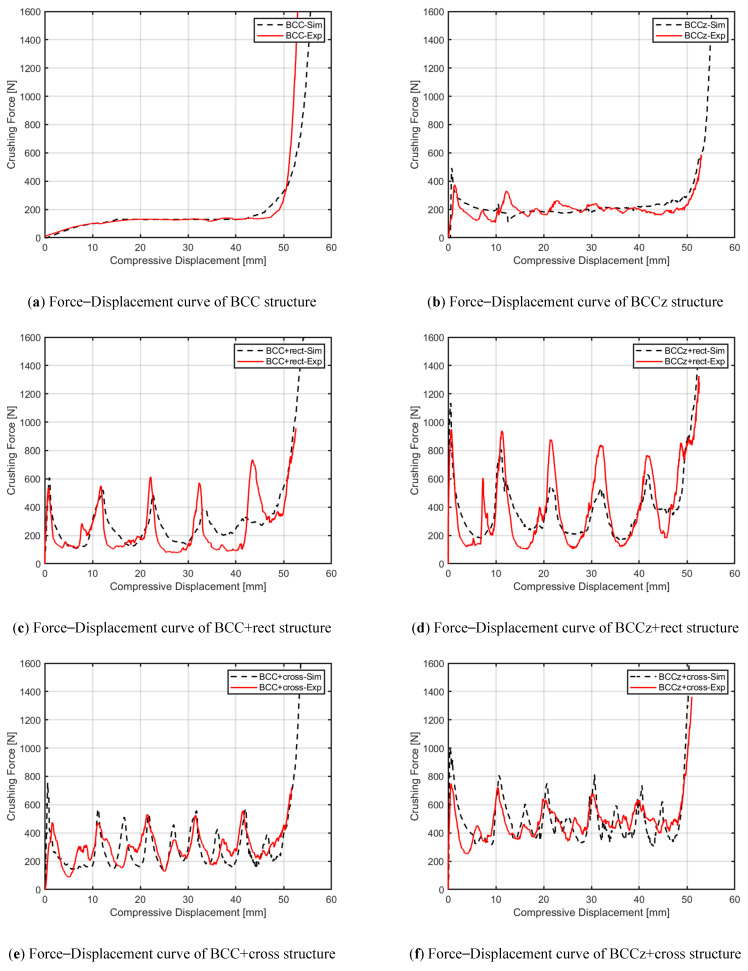
Comparison of crushing force curves between FEA and experiments.

The comparative analysis of crushing force curves, based on compressive displacement data derived from both finite element analysis and quasi-static compression experiments, is depicted in Figure 25. The results show a close agreement between the FEA and experimental data for all structures. Analogous to the outcomes observed in finite element analysis, the addition of vertical auxiliaries to the BCC structure was found to result in little increase in STD, coupled with an augmentation in energy absorption performance.

Specifically, when employing a cross-shaped configuration for the horizontal auxiliary, higher tension is applied on horizontal auxiliaries during the compression process than rectangular horizontal auxiliaries, which is validated in Figure 9 and Figure 10. This tension effect contributed to the structure’s ability to withstand higher loads.

**Figure 26:**
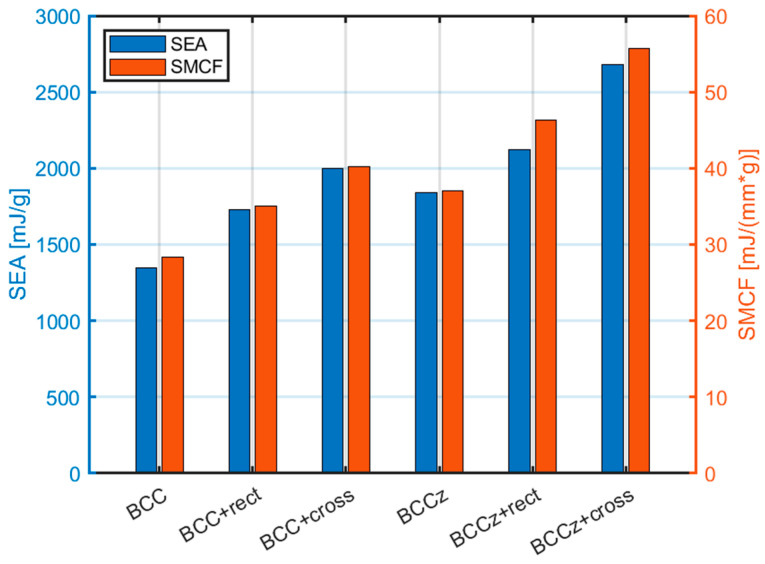
Comparison of SEA and SMCF for lattice structures.

In Figure 26, a graph illustrating the SEA and SMCF of each structure is presented as energy absorption indicators. The results demonstrate a consistent tendency of increasing SEA and SMCF values with the addition of vertical auxiliary struts to the lattice structure. Furthermore, the addition of horizontal aids also contributes to an improvement in both SEA and SMCF. Notably, the cross-shaped horizontal auxiliary struts exhibit a greater enhancement in performance compared to the rectangular-shaped auxiliary struts in terms of both SEA and SMCF. This observation highlights the superior energy absorption capabilities achieved by incorporating the cross-shaped horizontal and vertical auxiliary struts.

**Figure 27:**
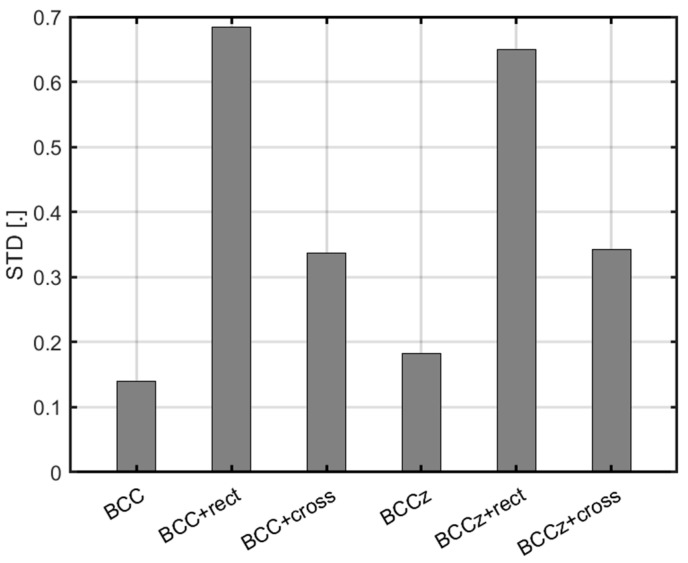
Comparison of STD for lattice structures.

In Figure 27, a graph illustrating the STD of each structure is presented. The optimal performance in STD is achieved in the structures without horizontal auxiliaries. However, the figure illustrates a notable improvement in energy absorption performance upon the addition of horizontal auxiliaries. The highest energy absorption performance is achieved when employing a cross-shaped horizontal auxiliary and vertical auxiliary

Upon the examination of Figure 27, it is substantiated that the cross-shaped horizontal auxiliary not only amplifies energy absorption but also yields superior STD performance compared to a rectangular horizontal auxiliary. Furthermore, the data affirms that the introduction of vertical auxiliaries has a negligible impact on the observed trends in STD performance.

## 7. Conclusions

In this study we aimed to enhance the energy absorbing ability of lattice structures by incorporating auxiliary struts into the well-established BCC lattice structure. The auxiliary struts were designed specifically as vertical and horizontal, with the horizontal auxiliary struts further categorized into rectangular and cross-shaped. FEA was employed to investigate the compression mechanism of both individual and entire structures, and to examine the corresponding alterations in energy absorption performance during compression.

The BCC lattice structure exhibits a characteristic behavior of overall bending and deformation during compression, resulting in a consistent crushing force. However, this behavior leads to deformation occurring at a relatively low crushing force, limiting its energy absorption capacity. On the other hand, when a horizontal auxiliary strut is added to the structure, it becomes capable of withstanding higher loads and absorbing greater amounts of energy through deformation. However, a challenge arises in terms of crushing force variability due to the layer-by-layer compressive mechanism. In this context, it has been determined that the cross-shaped horizontal auxiliary strut outperforms the rectangular auxiliary strut by offering higher load-bearing capacity with lower STD. This makes the cross-shaped auxiliary strut more suitable for energy absorption, as it reduces the variability of the crushing force and enhances its effectiveness.

The addition of horizontal and vertical auxiliary struts resulted in a significant increase in the energy absorption indicators, as determined in this study. The autonomously developed BCCz+cross structure exhibited substantial improvements compared to the conventional BCC structure, with a 417% increase in EA, 198% increase in SEA, 410% increase in MCF, and 196% increase in SMCF. However, it was observed that along with the enhanced energy absorption, there was an increase in STD, leading to a decrease in the variability of energy absorption. Consequently, further research is warranted to address and mitigate this issue.

This study presents a novel and efficient lattice structure design with superior performance in key aspects, e.g., strength, energy absorption, and weight reduction. The design has significant potential for industrial applications, particularly in aerospace and automotive sectors, where it can enhance crashworthiness and reduce overall weight. Its compatibility with advanced manufacturing techniques, such as additive manufacturing, ensures scalability and cost-effectiveness for real-world production. Future research could focus on material optimization and environmental durability to broaden its applicability further. This innovative design bridges the gap between advanced structural concepts and practical industrial needs, paving the way for sustainable engineering solutions.

## Figures and Tables

**Table 1:** Energy absorption indicators of 4 × 4 × 5 lattice structures (12 mm unit cell) using a quasi-static compression FE analysis.

Unit Cell Type	Mass [g]	EA [mJ]	SEA [mJ/g]	MCF [N]	STD [.]	SMCF [mJ/(mm × g)]
BCC	5.5	5.21 × 10^3^	9.47 × 10^2^	1.13 × 10^2^	0.1051	20.54
BCC+rect	7.0	1.17 × 10^4^	1.68 × 10^3^	2.43 × 10^2^	0.3801	34.74
BCC+cross	7.7	1.33 × 10^4^	1.73 × 10^3^	2.69 × 10^2^	0.2750	34.89
BCCz	6.3	1.03 × 10^4^	1.64 × 10^3^	2.08 × 10^2^	0.1396	32.95
BCCz+rect	7.8	1.69 × 10^4^	2.19 × 10^3^	3.47 × 10^2^	0.3834	45.10
BCCz+cross	8.4	2.23 × 10^4^	2.66 × 10^3^	4.74 × 10^2^	0.2396	56.41

**Table 2:** Energy absorption indicators of 4 × 4 × 5 lattice structures (12 mm unit cell) under quasi-static compression test.

Unit Cell Type	Mass [g]	EA [mJ]	SEA [mJ/g]	MCF [N]	STD [.]	SMCF [mJ/(mm×g)]
BCC	4.0	5.40 × 10^3^	1.35 × 10^3^	1.14 × 10^2^	0.1403	28.41
BCC+rect	6.5	1.13 × 10^4^	1.73 × 10^3^	2.27 × 10^2^	0.6848	35.03
BCC+cross	7.1	1.42 × 10^4^	2.00 × 10^3^	2.86 × 10^2^	0.3371	40.21
BCCz	5.3	9.76 × 10^3^	1.84 × 10^3^	1.96 × 10^2^	0.1819	37.07
BCCz+rect	7.7	1.63 × 10^4^	2.12 × 10^3^	3.57 × 10^2^	0.6491	46.37
BCCz+cross	8.4	2.25 × 10^4^	2.68 × 10^3^	4.68 × 10^2^	0.3424	55.77

**Figure A1:**
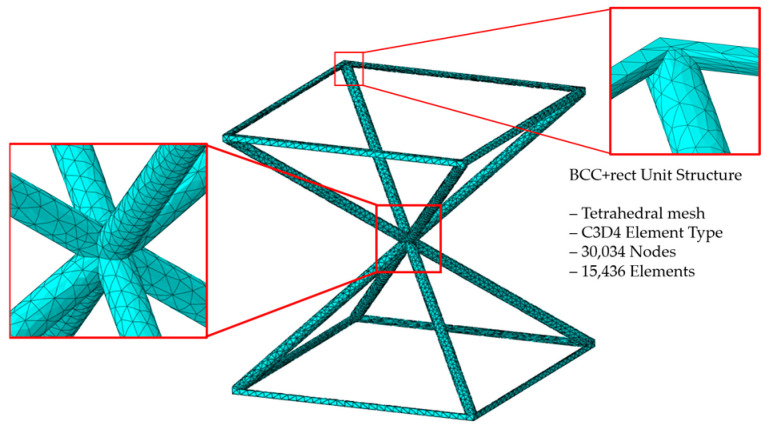
Finite element model of BCC+rect unit cell structure.

**Figure A2:**
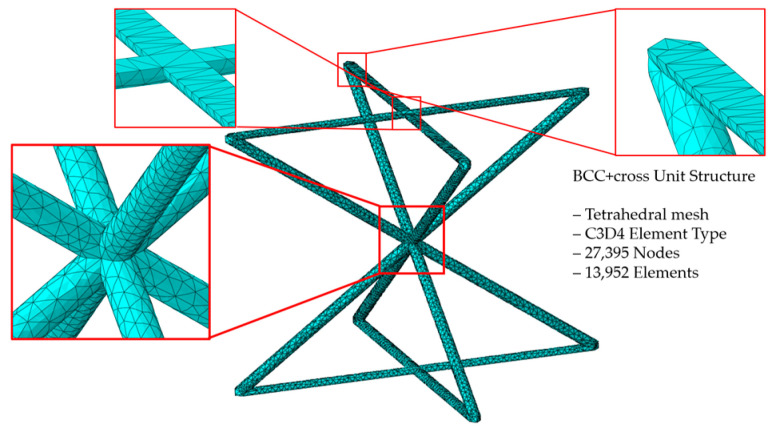
Finite element model of BCC+cross unit cell structure.

**Figure A3:**
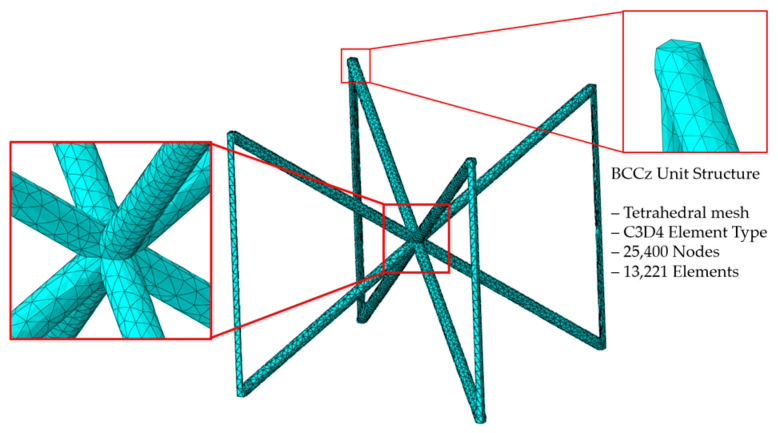
Finite element model of BCCz unit cell structure.

**Figure A4:**
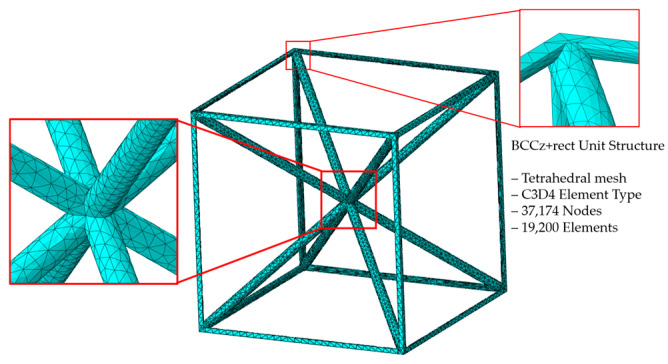
Finite element model of BCCz+rect unit cell structure.

**Figure A5:**
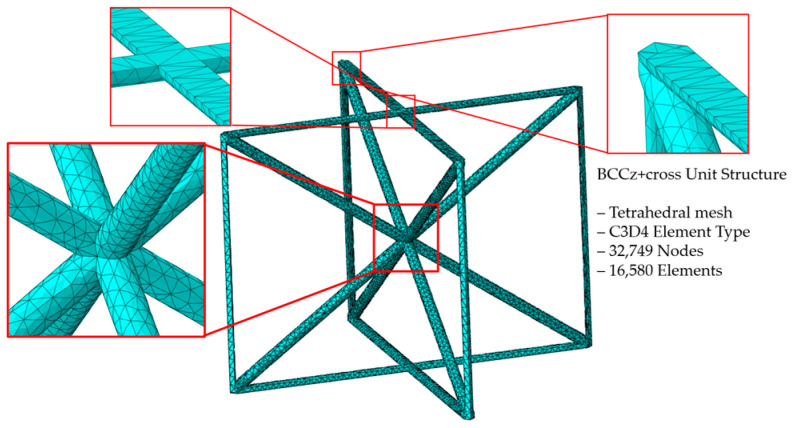
Finite element model of BCCz+cross unit cell structure.

**Figure A6:**
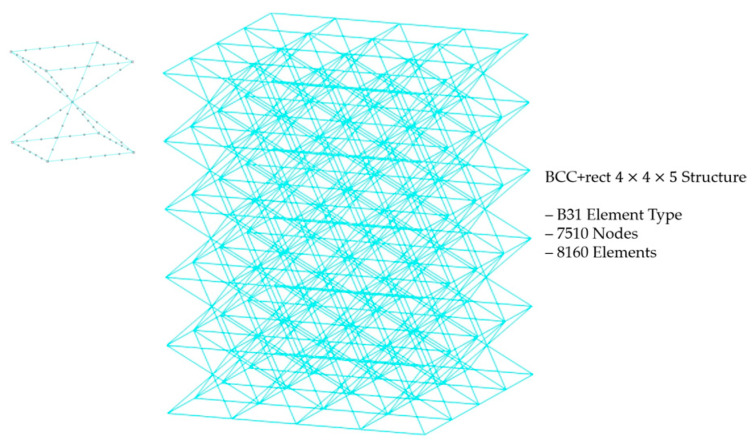
Finite element model of 4 × 4 × 5 BCC+rect lattice structure.

**Figure A7:**
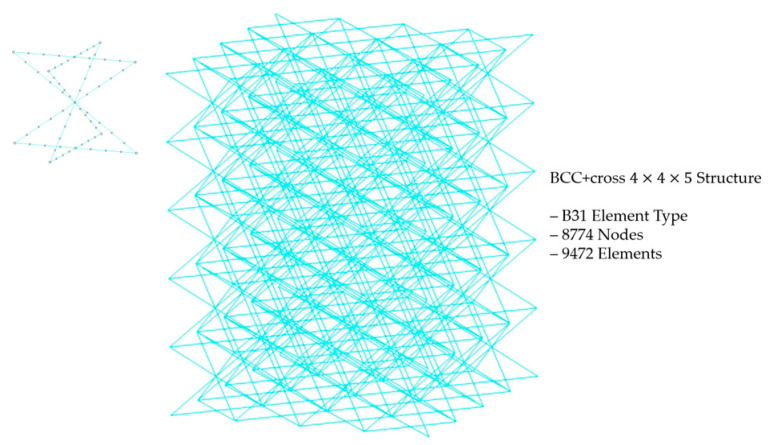
Finite element model of 4 × 4 × 5 BCC+cross lattice structure.

**Figure A8:**
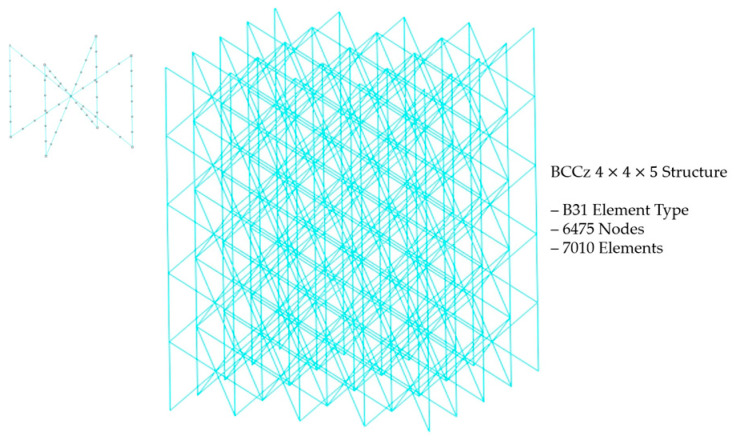
Finite element model of 4 × 4 × 5 BCCz lattice structure.

**Figure A9:**
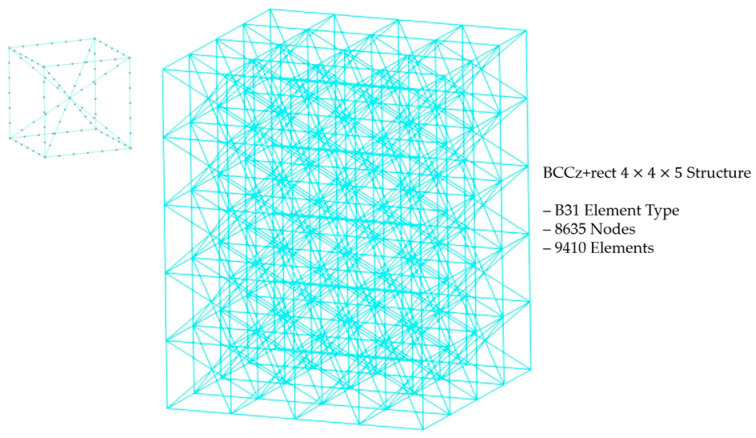
Finite element model of 4 × 4 × 5 BCCz+rect lattice structure.

**Figure A10:**
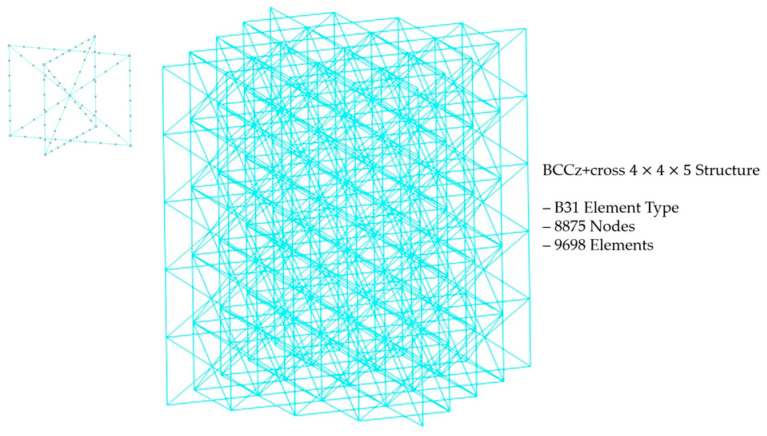
Finite element model of 4 × 4 × 5 BCCz+cross lattice structure.

**Figure A11:**
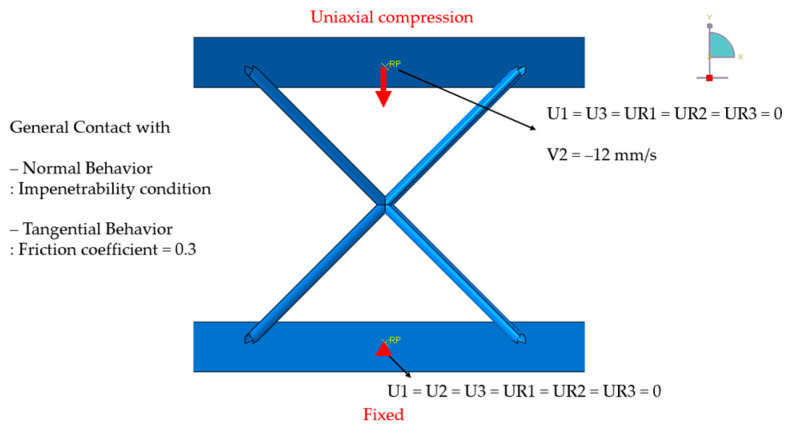
Boundary condition for quasi-static analysis of BCC unit cell structure under compressive loading.

**Figure A12:**
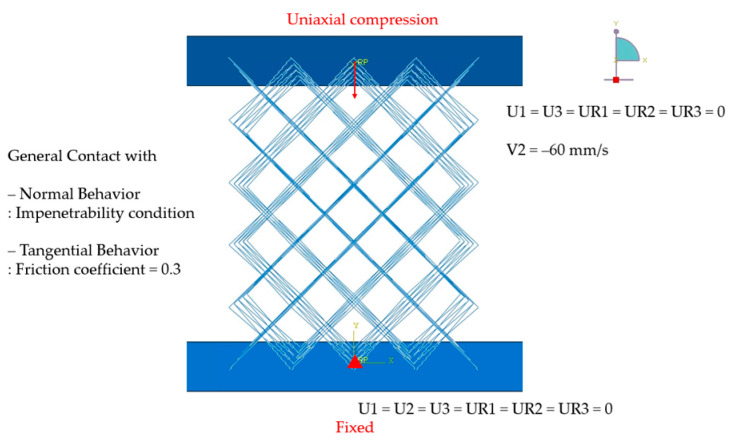
Boundary condition for quasi-static analysis of BCC 4 × 4 × 5 lattice structure under compressive loading condition.

## Data Availability

The original contributions presented in the study are included in the article, further inquiries can be directed to the corresponding authors.

## Abbreviations

BCC	Body-Centered Cubic
BCCz	BCC with Z strut
*EA*	Energy Absorption
EBM	Electron Beam Melting
FEA	Finite Element Analysis
*MCF*	Mean Crushing Force
*SEA*	Specific Energy Absorption
SLM	Selective Laser Melting
*SMCF*	Specific Mean Crushing Force
*STD*	Standard Deviation of Crushing Force
